# The Impact of Ang-(1-9) and Ang-(3-7) on the Biological Properties of Prostate Cancer Cells by Modulation of Inflammatory and Steroidogenesis Pathway Genes

**DOI:** 10.3390/ijms21176227

**Published:** 2020-08-28

**Authors:** Kamila Domińska, Karolina Kowalska, Kinga Anna Urbanek, Dominika Ewa Habrowska-Górczyńska, Tomasz Ochędalski, Agnieszka Wanda Piastowska Ciesielska

**Affiliations:** 1 Department of Comparative Endocrinology, Medical University of Lodz, Zeligowskiego 7/9, 90-752 Lodz, Poland; tomasz.ochedalski@umed.lodz.pl; 2 Department of Cell Cultures and Genomic Analysis, Medical University of Lodz, Zeligowskiego 7/9, 90-752 Lodz, Poland; karolina.kowalska1@umed.lodz.pl (K.K.); kinga.urbanek@umed.lodz.pl (K.A.U.); dominika.habrowska@umed.lodz.pl (D.E.H.-G.); agnieszka.piastowska@umed.lodz.pl (A.W.P.C.)

**Keywords:** angiotensin, RAS, prostate cancer, proliferation, migration, steroidogenesis, NF-kB

## Abstract

The local renin–angiotensin system (RAS) plays an important role in the pathophysiology of the prostate, including cancer development and progression. The Ang-(1-9) and Ang-(3-7) are the less known active peptides of RAS. This study examines the influence of these two peptide hormones on the metabolic activity, proliferation and migration of prostate cancer cells. Significant changes in MTT dye reduction were observed depending on the type of angiotensin and its concentration as well as time of incubation. Ang-(1-9) did not regulate the 2D cell division of either prostate cancer lines however, it reduced the size of LNCaP colonies formed in soft agar, maybe through down-regulation of the *HIF1a* gene. Ang-(3-7) increased the number of PC3 cells in the S phase and improved anchorage-independent growth as well as mobility. In this case, a significant increase in *MKI67*, *BIRC5,* and *CDH-1* gene expression was also observed as well as all members of the NF-kB family. Furthermore, we speculate that this peptide can repress the proliferation of LNCaP cells by NOS3-mediated G2/M cell cycle arrest. No changes in expression of *BIRC5* and *BCL2/BAX* ratio were observed but a decrease mRNA proapoptotic *BAD* gene was seen. In the both lines, Ang-(3-7) improved *ROCK1* gene expression however, increased *VEGF* and *NOS3* mRNA was only seen in the PC3 or LNCaP cells, respectively. Interestingly, it appears that Ang-(1-9) and Ang-(3-7) can modulate the level of steroidogenic enzymes responsible for converting cholesterol to testosterone in both prostate cancer lines. Furthermore, in PC3 cells, Ang-(1-9) upregulated *AR* expression while Ang-(3-7) upregulated the expression of both estrogen receptor genes. Ang-(1-9) and Ang-(3-7) can impact on biological properties of prostate cancer cells by modulating inflammatory and steroidogenesis pathway genes, among others.

## 1. Introduction

The literature confirms that selected components of the renin–angiotensin system (RAS) change in different types of tumors, including prostate cancer. Two opposing signaling pathways are described: The ACE1/Ang II/AT1 axis stimulating the cell proliferation, tumor cell motility and metastasis, and the ACE2/Ang-(1-7)/Mas axis, which can limit the cell divisions and reduce the invasive potential of cancer cells [1,2,3,4]. However, the complicated network of mutual dependencies in the discussed RAS includes much more bioactive peptide hormones, locally produced by the prostate and acting in the autocrine and paracrine pathways. There is some data on the impact of Ang III and Ang IV on the various properties of the presented prostate cancer cells, such as viability or migration potential [5,6,7,8]. Our previous research demonstrated that Ang IV decreases proliferation in the androgen-dependent cells but not an androgen-independent line [8]. Similarly, Ang III promoted cell migration of LNCaP to a much greater degree than DU-145 cells [7]. Ang-(1-9) and Ang-(3-7) are also interesting metabolites of the RA system, due to their opposite effect on the prostate epithelial cells. Ang-(1-9) was found to have mitogenic activity while Ang-(3-7) decreased the number of PNT1A cells in the phase G2/M of the cell cycle. Furthermore, our results suggested pro-mobility role of Ang-(1-9) and anti-migratory function of Ang-(3-7) in the in vitro human prostatic cell cultures [9]. Therefore, the present study examines whether Ang-(1-9) and Ang-(3-7) can also modulate the properties of prostate cancer cells for example by genes associated with cell cycle regulation and oxidative stress. All experiments variants were conducted on the two prostate cancer lines representing different growth rates, degree of aggressiveness and hormonal status in order to take into account the heterogeneity of this disease. Undoubtedly, the prostate cancer is an example of a hormone-dependent malignancy. The latest research results state that not only androgens but also estrogens play an important role in the control of prostate cancer growth, differentiation and metastasis [10,11,12]. Therefore, we evaluated the changes in the mRNA and protein levels of *AR*/AR, *ESR1*/ERα, *ESR2*/ERβ, and expression of enzymes involved in the biosynthesis of sex hormones: *CYP17A1*, *CYP19A1*, *HSD17B*, and *HSD3B1*.

## 2. Results

### 2.1. Influence of Ang-(1-9) and Ang-(3-7) on Metabolic Activity of Prostate Cancer Cells

After 48-h incubation, Ang-(1-9) increased MTT reduction in LNCaP cells at all tested concentrations, while only the lowest concentration was effective in PC3. With shorter incubation time (24 h), only selected concentrations of Ang-(1-9) (0.1 nM, 100 nM, and 1000 nM) induced significant MTT reduction in androgen-sensitive cells. In the case of androgen-insensitive prostate cancer cells, Ang-(1-9) temporarily reduced metabolic activity.

**Figure 1:**
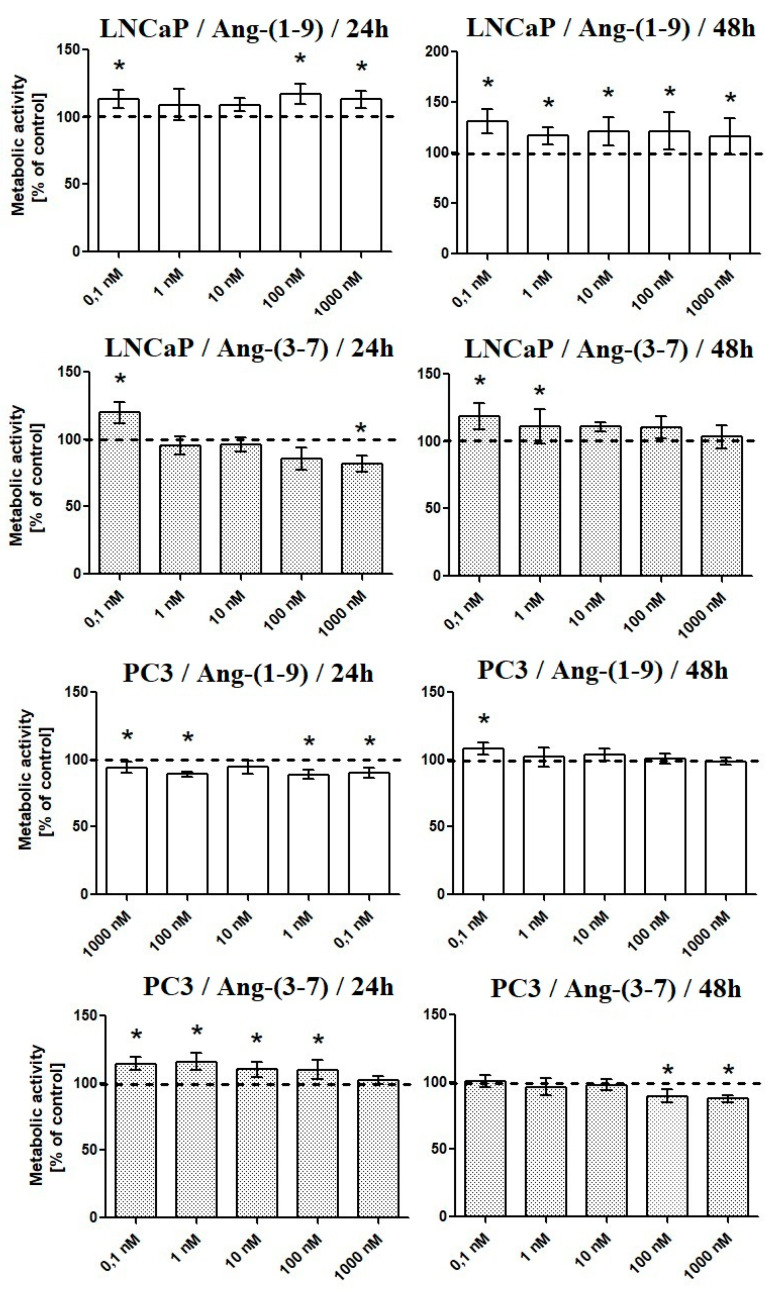
The MTT test results after incubation of prostate cancer cells (LNCaP, PC3) with various concentrations of Ang-(1-9) and Ang-(3-7) (0.1 nM–1000 nM) (mean ± SD; one-way ANOVA with post-hoc Dunnett’s test: * *p* < 0.05).

**Figure 2:**
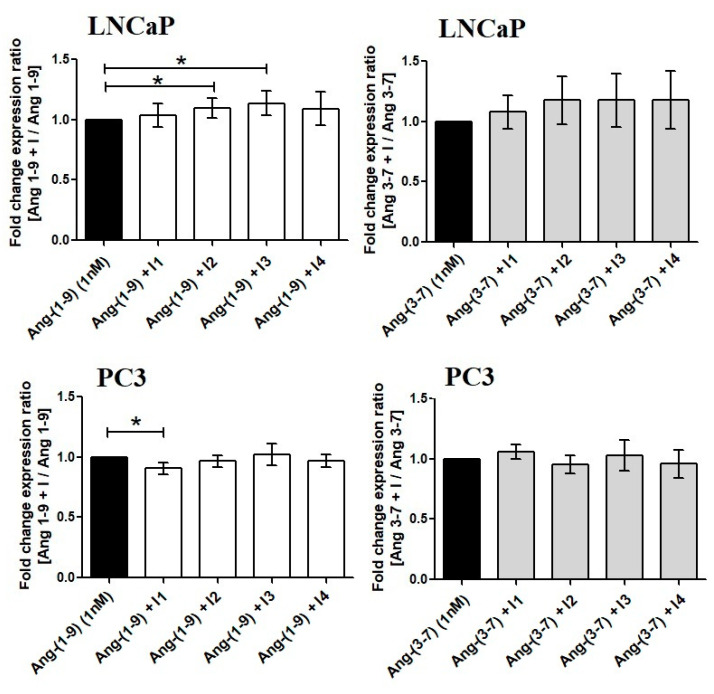
The MTT test results showing the impact of angiotensin receptor inhibitors on Ang-(1-9) and Ang-(3-7) (1 nM) activity in prostate cancer cells: LNCaP, and PC3. (I1: AT1 inhibitor—losartan; I2: AT2 inhibitor—PD123319; I3: AT1–7/MAS inhibitor—A779; I4—AT4/IRAP inhibitor—HFI142; 1000 nM) (Dunnett’s test; * *p* < 0.05).

Ang-(3-7) stimulated the metabolic activity of LNCaP cells at the two lowest concentrations (0.1 and 1 nM) and inhibited PC3 cells at the two highest concentrations (48 h). Following 24-h incubation, Ang-(3-7) increases the MTT reduction in PC3 (0.1–100 nM) and LNCaP (0.1 nM). At the highest concentration, was neutral (PC3) or decreased (LNCaP) cell metabolic activity (Figure 1). None of the angiotensin receptor inhibitors failed to reverse the stimulating effect of Ang-(3-7) in LNCaP cells. On the other hand, the AT2 and AT1-7/MAS receptor antagonists (PD 123,319 and A779) poorly but significantly intensified the effect of Ang-(1-9) in LNCaP cells (Figure 2).

### 2.2. Influence of Ang-(1-9) and Ang-(3-7) on Cell Proliferation of Prostate Cancer Lines

**Figure 3:**
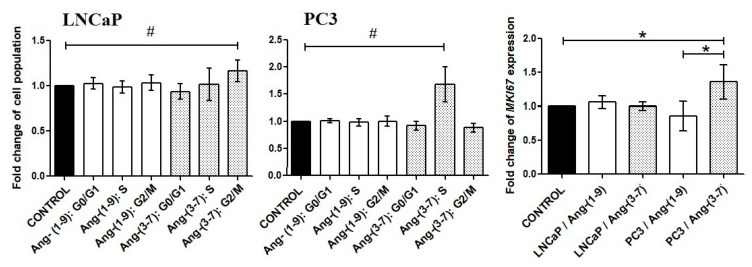
The Muse Cell Cycle Assay results, following incubation (48 h) of prostate cancer cells (LNCaP, PC3) with Ang-(1-9) and Ang-(3-7) at concentration 1 nM (mean ± SD; one-way ANOVA with post-hoc Dunnett’s test: # *p* < 0.05 or Tukey’s test: * *p* < 0.05).

Incubation of prostate cancer cells with Ang-(1-9) did not affect the proportion of cells in particular phases of the cell cycle. In contrast, Ang-(3-7) increased the number of PC3 cells in the S phase, in which DNA is replicated, and LNCaP cells in the G2/M phase. The increase of LNCaP cell population at the G2/M phase was accompanied by a decrease of cell population in the G1 phase of the cell cycle; however, this was statistically insignificant. Only in the case PC3 cells, was the *MKI67* gene upregulated, which codes a cellular marker for proliferation (Figure 3).

**Figure 4:**
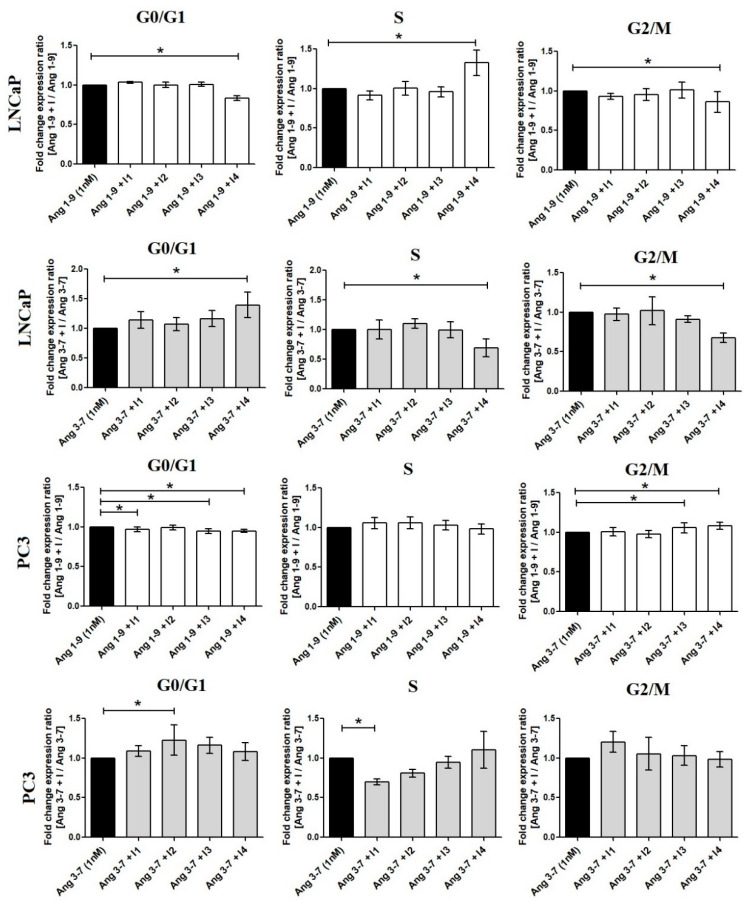
The Muse Cell Cycle Assay results showing the impact of angiotensin receptor inhibitors on Ang-(1-9) and Ang-(3-7) (1 nM) activity in prostate cancer cells: LNCaP and PC3. (I1: AT1 inhibitor—losartan; I2: AT2 inhibitor—PD123319; I3: AT1–7/MAS inhibitor—A779; I4—AT4/IRAP inhibitor—HFI142; 1000 nM) (Dunnett’s test; * *p* < 0.05).

Experiments with selective inhibitors of angiotensin receptors suggested that AT4/IRAP can play an important role in LNCaP cells. In PC3 we observed that the AT1 and AT2 inhibitors partially reverse the effect of Ang-(3-7) (Figure 4).

### 2.3. Influence of Ang-(1-9) and Ang-(3-7) on Anchorage-Independent Cell Growth Ability and Cell Mobility of Prostate Cancer Lines

**Figure 5:**
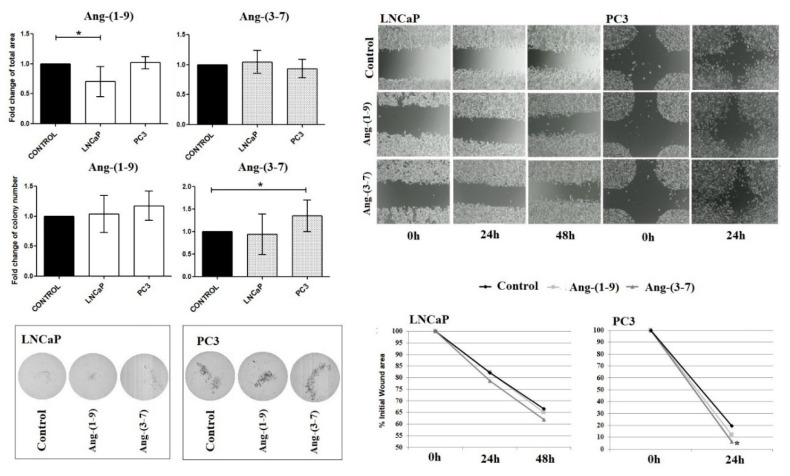
The Soft Agar Colony Formation Assay and Wound Healing Assay results after incubation of prostate cancer cells (LNCaP, PC3) with Ang-(1-9) and Ang-(3-7) at a concentration of 1 nM (mean ± SD; one-way ANOVA with the post hoc Dunnett’s test: * *p* < 0.05).

As shown in Figure 5, Ang-(1-9) reduces colony sizes of the LNCaP cells in soft agar, while the number of colonies remained unchanged. On the contrary, Ang-(3-7) stimulated the number of PC3 colonies formed in the agarose gel compared to controls, but did not affect colony size. Furthermore, Ang-(3-7) increased the mobility of prostate cancer cells; however, significant results were only observed for the PC3 line.

### 2.4. Influence of Ang-(1-9) and Ang-(3-7) on mRNA Level of Angiotensin Receptors Gene

**Figure 6:**
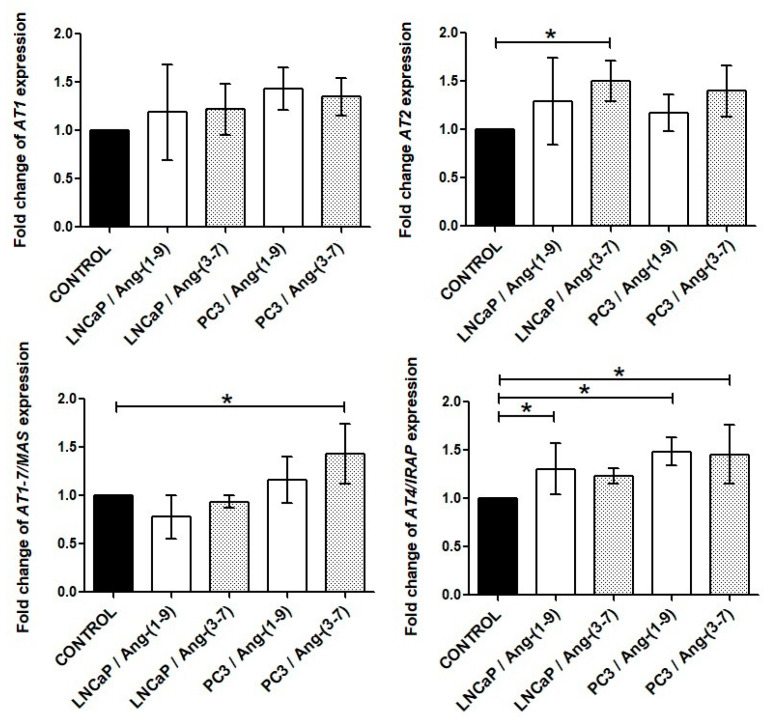
The RT-qPCR results about expression of angiotensin receptor genes in the prostate cancer lines (LNCaP and PC3) after exposure to Ang-(1-9) or Ang-(3-7) at a concentration of 1 nM for 48 h (mean ± SD; Tukey’s test: * *p* < 0.05)

In the case of the LNCaP line, both the *AT2* and *AT4/IRAP* receptors exhibited significantly greater expression after treatment with Ang-(3-7) and Ang-(1-9), respectively. More changes in angiotensin receptor level could be observed in the PC3 line: Ang-(1-9) stimulated the expression of the *AT4/IRAP* receptor while Ang-(3-7) enhanced the mRNA level of *AT1-7/MAS* and *AT4/IRAP* receptors (Figure 6).

### 2.5. Influence of Ang-(1-9) and Ang-(3-7) on mRNA Level of Inflammatory Pathway Genes

**Figure 7:**
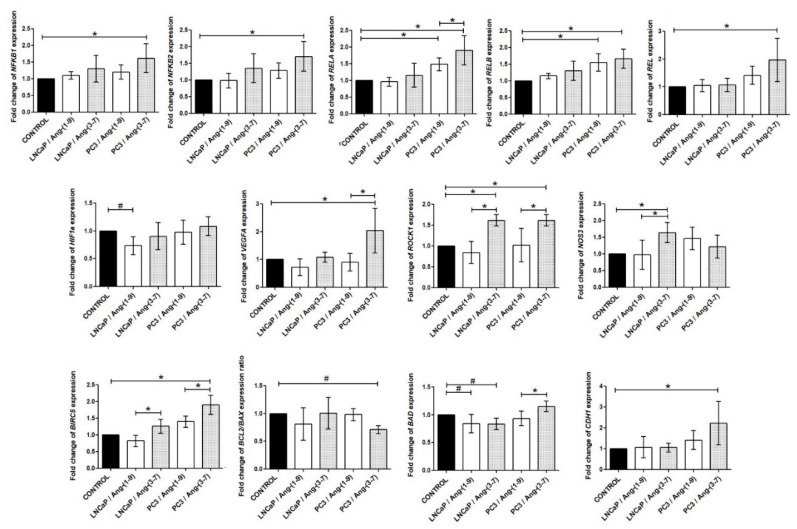
RT-qPCR analysis of the expression of inflammatory and apoptotic pathway genes in the prostate cancer lines (LNCaP and PC3) after exposure to Ang-(1-9) or Ang-(3-7) at a concentration of 1 nM for 48 h (mean ± SEM; Tukey’s test: * *p* < 0.05; Dunnett’s test: # *p* < 0.05).

Firstly, we investigated the gene expression profiles of the NF-κB pathway in prostate cancer lines after angiotensin treatment. The androgen-sensitive prostate cancer cell line did not show significant changes in any of the members of the NF-kB family. In PC3 cells, both angiotensins stimulated expression of *RELA* and *RELB*, but *NFKB1*, *NFKB2,* and *REL* up-regulated only by Ang-(3-7). The results for the remaining analyzed inflammatory pathway genes are presented below. The expression level of the *NOS3* gene was stimulated by Ang-(3-7) but only at LNCaP cells. This variant also increased expression of the *ROCK1* gene. In PC3 cells, the level of the *VEGFA* and *ROCK1* mRNA was increased after incubation with Ang-(3-7) but not Ang-(1-9). The *HIF1a* gene was down-regulated in LNCaP cells only by Ang-(1-9). It is worth noting that *BCL2/BAX* expression ratio was found to be significantly decreased in PC3 cells treated with Ang-(3-7). On the other hand, the *BIRC5* and *CDH-1* mRNA was up-regulated. LNCaP cells demonstrated a decreased level of *BAD* expression after treatment with both Ang-(1-9) and Ang-(3-7) (Figure 7).

### 2.6. Influence of Ang-(1-9) and Ang-(3-7) on mRNA Level of Steroidogenesis Pathway Genes

**Figure 8:**
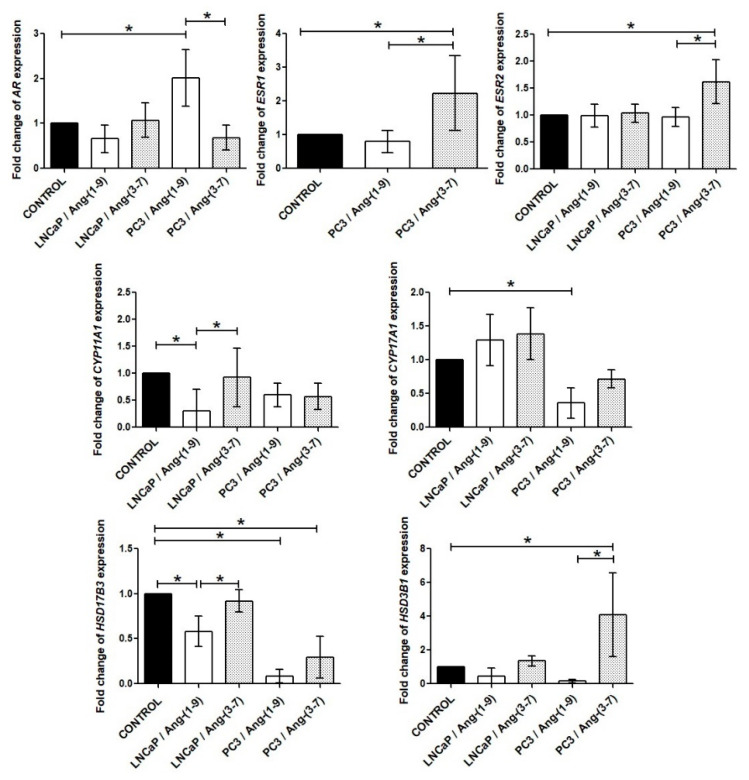
RT-qPCR analysis of steroidogenesis pathway gene expression in the prostate cancer lines (LNCaP and PC3) after exposure to Ang-(1-9) or Ang-(3-7) at a concentration of 1 nM for 48 h (mean ± SD; Tukey’s test: * *p* < 0.05).

**Figure 9:**
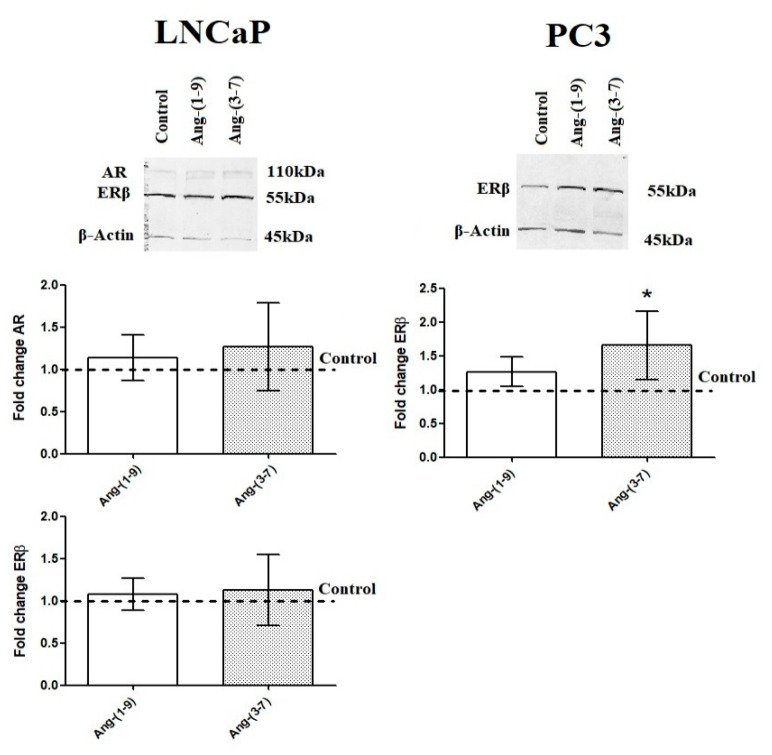
Western blot analysis of androgen receptor (AR) and ERs level in the prostate cancer lines (LNCaP and PC3) after exposure to Ang-(1-9) or Ang-(3-7) at a concentration of 1 nM for 48 h (mean ± SD; Tukey’s test: * *p* < 0.05).

It is worth emphasizing that androgen receptor (AR) has been expressed in both prostate cancer cells but in PC3 line is many times lower than in LNCaP. Moreover, estrogen receptor 2 (*ESR2*/ERβ) is more highly expressed in PC3 cells than in LNCaP. The estrogen receptor 1 (*ESR1*/ERα) has a low level of expression in PC3 line and almost undetectable in LNCaP cells [13]. The LNCaP cells did not show significant changes in any of steroid hormone receptors after incubation with tested angiotensins. In PC3 cells, Ang-(1-9) upregulated *AR* expression while Ang-(3-7) upregulated the expression of both estrogen receptor genes (*ESR1* and *ESR2*) (Figure 8). The Western blot method confirmed the stimulating effect of Ang-(3-7) on the level of ERβ in PC3 cells. AR and ERα detection failed in the above-mentioned prostate cancer line. The both angiotensin peptides did not change the levels of AR and ERβ in the LNCaP line (Figure 9).

We also observed changes in the mRNA of enzymes involved in steroid hormone biosynthesis after 48 h incubation with tested angiotensins. *CYP11A1* and *CYP17A1* gene expression was inhibited by Ang-(1-9) in LNCaP and PC3 cells, respectively. The level of *HSD17B3* mRNA was also decreased by Ang-(1-9) in the both tested prostate cancer cell lines. On the other hand, the expression of *HSD3B1* gene was strongly stimulated by Ang-(3-7), but only in PC3 cells (Figure 8).

## 3. Discussion

The activity of the local renin–angiotensin system (RAS) in the prostate has been documented by immunohistochemistry (IHC) and polymerase chain reaction (PCR) [14]. The RAS is known to play an important role in the pathophysiology of prostate gland, including cancer development and progression [1,2,3,14]. Ang-(1-9) and Ang-(3-7) are one of the less known but biologically active peptides of the RAS. Our earlier research presented the influence of these angiotensins on viability and proliferation, migration, and invasion of epithelial prostate cells. In PNT1A cells Ang-(1-9) and Ang-(3-7) exerted opposite effects. The first angiotensin showed mitogenic and pro-movement properties while the other had no proliferative activity and showed anti-migratory activity [9].

Our present findings indicate that Ang-(1-9) and Ang-(3-7) have different effects in epithelial cells and cancer cells of the prostate, and that androgen-sensitive and androgen-insensitive prostate cancer cells reacted differently according to the kind of tested peptide. Both peptides can accelerate, and sometimes weaken, the metabolic activity of prostate cancer cells. Their effect varies greatly, depending on the angiotensin concentration and time of incubation. The increase in MTT reduction observed in LNCaP cells after 48 h incubation with 1 nM Ang-(1-9) is probably not mediated by AT2 because its inhibitor intensified the effect of the peptide. PC3 cells treated with Ang-(1-9) plus losartan presented lower absorbance than cells incubated only with Ang-(1-9), which confirms the proproliferative function of AT1 receptor [1].

Furthermore, it appears that both angiotensins can regulate the mRNA levels of angiotensin receptors. Ang 3-7 improved the expression of the *AT2* receptor in LNCaP cells and increased the level of *AT1-7/MAS* and *AT4/IRAP* in PC3. The second of the tested peptides upregulated the expression of *AT4/IRAP* in both prostate cancer cell lines. As previously demonstrated, other system effector peptides, for example Ang II, Ang III, Ang IV, or Ang-(1-7), also have the ability to modulate angiotensin receptors expression [7,8,13,15]. In the epithelial prostate cells, Ang-(3-7) upregulated expression of *AT1-7/MAS*, as in the PC3 line, but down-regulated the level of receptor *AT4/IRAP*. The mRNA level of all known receptors for angiotensin was reduced by Ang-(1-9) in PNT1A cells [9].

Neither prostate cancer line demonstrated an increase in the number of cells in S phase after incubation with Ang-(1-9) (1 nM) as was the case with PNT1A cells. On the other hand, we noted that Ang-(3-7) stimulates expression of the proliferation marker *MKI67* in PC3, and this was accompanied by an increase in the cell population in the replication phase. Curiously, despite the upregulation of survivin (*BIRC5*), a slight diminished *BCL2/BAX* ratio was observed. In LNCaP cells, Ang-(3-7) raised the number of cells in the phase M/G2. We speculate that this peptide can repress the proliferation of LNCaP cells by NOS3-medieted G2/M cell cycle arrest. No changes in expression of *BIRC5* and *BCL2/BAX* ratio were observed, and the mRNA level of the proapoptotic *BAD* gene decreased, which seems to exclude apoptosis. Meanwhile in the prostate epithelial cells, Ang-(3-7) decrease the number of cells in G2/M phase of the cell cycle [9].

Experiments with selective inhibitors of angiotensin receptors suggest that AT4/IRAP mediates the activities of Ang-(3-7) in the LNCaP line. The ability of Ang-(3-7) to interact with the AT4/IRAP receptor has already been described in the scientific literature. Handa et al. (1999) presented that Ang-(3-7) inhibited action of Ang-(1–7) at the proximal tubule AT4-receptor site [16]. The AT1 receptor, possibly also AT2, is responsible for the increase in the number of PC3 cells in the S phase. The ability of PC3 cells to survive and grow without contact with the ECM was also improved after incubation with Ang-(3-7). We observed an increased number of colonies formed on the agarose gel compared to the control group but Ang-(3-7) did not affect colony size. The colony size was inhibited by Ang-(1-9) in androgen-sensitive cells. Our previous research has shown that Ang-(1-7) decreased the growth of the LNCaP, but not PC3 cell line in soft agar [13]. There is therefore a presumption that Ang-(1-9) may partially have been converted to Ang-(1-7) and thus decrease the anchorage-independent growth in LNCaP. Reminding, Ang-(1-9) can be converted to Ang-(1-7) by ACE or neutral endopeptidase (NEP).

It is possible that the reduction in colony size may be associated with the down-regulation of the *HIF1a* gene. The role of HIF1a in human cancers is still under intensive investigation, but HIF1a has been implicated in over 60 target genes, many of which are strongly activated in tumors. Furthermore, some results suggest that downregulation of HIF1a may decrease the proliferation and invasive ability of various types of cancer cells. The drugs that indirectly affect the HIF1a pathway and HIF1a inhibitors might be a good alternative in the adjuvant therapy of certain cancers [17,18,19].

In epithelial prostate cells, Ang-(1-9) has pro-migratory properties while Ang-(3-7) prevents migration. Furthermore, we noted Ang-(1-9) increase the level of *VEGFA* and *HIF1a*, while Ang-(3-7) inhibited the expression of these genes in PNT1A cells [9]. However, Ang-(3-7) increased the migration of prostate cancer cells (statistical significance only for PC3) while Ang-(1-9) had no significant effect. In both lines, Ang-(3-7) improved *ROCK1* gene expression while increased *VEGFA* mRNA was only seen in the PC3 cells. ROCK1 is a critical regulator of the cytoskeletal dynamics that control cell shape, motility, and colonization potential. Genetic variants in RhoA and ROCK1 genes have been suggested as susceptibility factors for prostate cancer development [20]. Immunohistochemistry staining presented higher ROCK1 levels in prostate cancer than in the normal epithelium [21,22]. Higher levels of ROCK1 were associated with prostate tumor stage, and Gleason grade, positive nodal stage, and poor prognosis [22,23]. The knockdown of rho as sociated protein kinase genes (ROCK) reduced the migration and invasion of PC3 and DU-145 cells in vitro, and promoted metastasis in a mouse model in vivo [24]. Interestingly, pharmacological inhibitors of the Rho pathway reportedly block angiogenesis. Indeed, some studies suggest that the Rho/ROCK pathway is involved in various aspects of formation of new blood vessels and that it is reportedly essential for VEGF-dependent migration, survival, and permeability [25,26].

Furthermore, an androgen-insensitive prostate cancer cell line showed significant changes in NF-kB family genes, while those in LNCaP showed stable, invariant expression. In the case of PNT1A cells, the impact of both peptides on NF-kB expression was also very limited. The significant difference was observed only for the *REL* gene in cells incubated for 48 h with Ang-(1-9) [9]. In our previous studies, we also showed that Ang-(1-7) can increase *NFKB1* and NFKB2 mRNA in prostate cancer cells [13]. An increase in the above-mentioned genes was observed in PC3 cells treated with Ang-(1-9), but not in LNCaP. Also, the second of the peptides tested (Ang-(3-7)) upregulated the expression of all members of the NF-kB family only in the PC3 cells. The NF-kB family is a central signaling group of factors involved in the development and progression of human cancers as well as in the acquisition of a hormone-resistant phenotype in highly-aggressive malignancies. Generally, decreasing androgen levels or blocked androgen receptor signals are associated with an increase of these inflammatory markers [27,28,29,30].

The development of prostate cancer is conditioned by the hormonal state of the body. The RAS signal transduction pathways can interact with sex hormone receptors similarly to the growth factors. Some studies report that the expression and function of individual elements of local RAS may be regulated by sex hormones. For example, AT2R expression in the aorta correlated with an alteration in testosterone levels. In males, castration significantly upregulated AT2 mRNA whereas increased androgen levels by DHT in females significantly decreased AT2R expression [31]. Our earlier research demonstrated that the non-genomic effect of testosterone, but not 17β-estradiol, alters the effects of Ang-(1-7) on the activity of PTKs in prostate cancer cells. This phenomenon was related to the effect of testosterone on the interaction between Ang-(1-7) and AT2 receptor [32]. Furthermore other studies reported that the level of the sex steroid receptors may be regulated by angiotensins. The expression of sex- hormone receptors is essential for the growth, recurrence and metastasis of gynecological cancers. Our previous study showed that Ang II and Ang-(1-7) might modulate the expression of androgen and/or estrogen receptors in prostate cancer cells. Ang II treatment significantly increased *AR* expression in LNCaP cells but insignificantly decreased it in PC3 cells [33]. After exposure to Ang-(1-7), a significant up-regulation of *AR* expression was observed in LNCaP cells while a significant down-regulation was seen in the DU-145 and PC3 cells. Furthermore, decreased expression of *ESR1* and a noticeable increase in *ESR2* mRNA was observed in all prostate cancer cells [13].

In this study, we also attempted to identify the relationship between Ang-(1-9) or Ang-(3-7) and the levels of sex steroid receptors and steroidogenic enzymes in the pathway from cholesterol to active androgens. Organs previously considered as the targets of sex steroids, such as the prostate, are also capable of local steroidogenesis, either from blood-borne prohormones or de novo biosynthesis from cholesterol. Independent sex hormone secretion is one of the key tactics used by cancer cells to overcome typical androgen deprivation therapies, such as pharmacological suppression of the hypothalamic–pituitary–gonadal axis or the usage of steroid receptor antagonists. These treatments often reduce the growth and metastatic potential of tumors; however, they are only effective in the short-term. The emergence of resistance to castration is an inevitable, and lethal, consequence. Therefore, the importance of these locally-produced steroids is a subject of study in a variety of disciplines, not only reproductive physiology but also oncology [34,35].

The enzymes CYP11A1, CYP17A1, HSD17B3, and HSD3B1 are known to play substantial roles in the conversion of cholesterol to testosterone. The human *CYP11A1* gene encodes the cholesterol side-chain cleavage enzyme (P450scc), which mediates the first step of steroidogenesis. This mitochondrial enzyme catalyzes the conversion of cholesterol to pregnenolone. The human *CYP17A1* gene encodes steroid 17-alpha-hydroxylase (P450c17); it is required for the production of androgenic and estrogenic sex steroids by converting pregnenolone and progesterone to dehydroepiandrosterone (DHEA) and androstenedione, respectively, in a two-step reaction. The human *HSD17B3* gene encodes 17β-hydroxysteroid (17β-HSD3) dehydrogenases, which catalyze the conversion of weakly androgenic androstenedione to testosterone. Finally, the human *HSD3B1* gene encodes 3β-hydroxysteroid dehydrogenase-1 (3βHSD1) which converts dehydroepiandrosterone into dihydrotestosterone [36].

Several polymorphisms in sex steroid metabolic pathways have been shown to be associated with the risk and progression of prostate cancer. For example, Kumazawa et al. (2004) suggest that microsatellite polymorphism of the steroid hormone synthesis gene *CYP11A1* may have a significant influence on the development of advanced prostate cancer [37]. Stanford et al. (2002) noted that the CYP17 A2/A2 genotype predicts susceptibility to prostate cancer in Caucasian men with a family history of the disease [38]. The results regarding the relationship between rs743572 gene polymorphism and prostate cancer are divergent and appear to depend on the nationality and age of the patients [39]. However, the use of CYP17A1 inhibitors (e.g., abiraterone) has developed as an important therapeutic alternative in castration-resistant prostate cancer (CRPC) [40]. Margiotti et al. (2002) associated the G289S single nucleotide polymorphism in the HSD17B3 gene with risk for prostate cancer in Italian men. Wu et al. 2015 found that patients with a heterozygous variant (1245A>C) HSD3B1 gene develop CRPC at an increased frequency. However, no differences in survival time or mortality risk were observed [41]. Importantly, 3β-HSD converted steroidal CYP17A1 inhibitors, including abiraterone to active metabolites with androgen-like agonistic effects [42,43].

We observed that the mRNA level of *CYP11A1* and *HSD17B3* was reduced in LNCaP cells incubated with Ang-(1-9). In the androgen-insensitive line, Ang-(1-9) decreased the mRNA level of all tested steroidogenic enzymes, but only *CYP17A1* and *HSD17B3* significantly. A deficiency of these enzymes results in reduced production of steroid hormones, especially androgens. There are studies that show that HIF1a can regulate the expression of several steroidogenic enzyme genes [44]. It is important to note that Ang-(1-9) downregulated *HIF1α* in androgen-sensitive but not androgen-insensitive prostate cancer cells. On the other hand, Ang-(1-9) treatment resulted in a lower level of steroidogenic enzymes with a compensatory increase in *AR* expression in the PC3 line, which was not seen in the LNCaP line. Ang-(3-7) downregulated mRNA *CYP17A1* and strongly upregulated *HSD3B1* gene expression in the PC3 cells. 3βHSD1 enzymatic activity is essential for the synthesis of potent androgens from adrenal precursor steroids in prostate cancer.

Interestingly, Ang-(3-7) significantly increased the expression of both estrogen receptor genes *ESR1* (ERα) and ESR2 (ERβ). The expression of sex-steroid receptors in PC3 cells dramatically favored estrogen signaling because *ESR2* is expressed more strongly than *AR* or *ESR1* (data not shown). The androgen metabolites generated by the aromatase-independent enzymes can also activate estrogen receptors. For example, 5α-Androstane-3β,17β-diol (3β-diol) which is generated from DHT, shows both androgenic and substantial estrogenic activities [45]. 3β-diol is a selective, potent, high-affinity full agonist of the ERβ. Studies suggest that ERβ signaling may act as a suppressor in prostate growth and play both anti-proliferative and apoptotic roles in the prostate. However, in our study, we noted an increase in the number of cells in the S phase of the cell cycle as well as an increase in *MKI67* expression in PC3 cells after incubation with Ang-(3-7). The expression of the *ESR1* gene in both LNCaP and PC3 cells was very low or even undetectable. On the other hand some results indicate that 3a-diol is inactive at AR, but induces prostate growth. However, it is worth remembering that 3b-diol is potentially a precursor of DHT via different pathways, such as HSD17B6 enzyme. [43,46].

Earlier, PC3 cells were considered to be androgen receptor negative (AR-). However, other studies have provided evidence that both DU-145 and PC3 cells express low, but detectable, levels of AR mRNA and protein [47]. Furthermore, many studies also present the effect of DHT on prostate cancer cells through non-androgen receptor pathway. For example Song et al. (2014) showed that dihydrotestosterone can enhance the proliferation of DU-145 and PC3 cells through STAT5 activation [48]. Finally, 3b-diol and testosterone can be metabolized via CYP19 (aromatase P450) into the potent estrogen, estradiol-17β (E2). In recent years there has been an influx of information about influence of estrogens contributed to the development and progression of prostate cancer [12].

Standard theory is that ERα drives proliferation in response to E2 while ERβ is growth inhibitory in prostate tissue. On the other hand, it has been confirmed that some variants of ERβ, such as ERβ2 and ERβ5, can act as stimulators of prostate cancerogenesis by improving cell proliferation, migration, and invasion under specific circumstances [10,11,12,32]. Given the huge disparities between level of androgen receptor and estrogen receptors in PC3 cells it can be assumed that ERα, may be also ERβ, mediates the pro-proliferative properties of Ang-(3-7) in this cell line. Clearly more research is needed to confirm this theory.

Collectively, these findings suggest that Ang-(1-9) and Ang-(3-7) can impact on biological properties of prostate cancer cells by the modulation of inflammatory and steroidogenesis pathway genes among others. Moreover, both Ang- (1-9) and Ang- (3-7) showed the ability to induce changes in the level of steroid receptors (AR and ERs) in aggressive prostate cells. This suggests that RAS and these peptides may be contributing to the transition from hormone-dependent prostate cancer to hormone-refractory disease.

## 4. Materials and Methods

### 4.1. Cell Culture and Reagents

We used one slow growth, androgen-sensitive prostate cancer cell line LNCaP (DSMZ, Braunschweig, Germany) and invasiveness, androgen-insensitive cell line PC3 (ECACC). In 2014, the lines were authenticated by short-tandem repeat (STR) DNA profiling (LGC Standards Cell Line Authentication Service, Germany). Human prostate cancer cells were maintained as a traditional monolayer culture in RPMI (Gibco, Thermo Fisher Scientific Inc, Waltham, MA, USA) with 10% heat-inactivated fetal bovine serum (FBS) and standard supplements, such as: sodium pyruvate, L-glutamine, HEPES buffer and antibiotics (PSN). Ang-(1-9) (no. H-5038) and Ang-(3-7) (no. H-6965) were purchased from Bachem (Bubendorf, Switzerland), while angiotensin receptor inhibitors were purchased from TOCRIS (Bristol, UK): losartan (AT1 antagonist, no. 3798), PD123319 (AT2 antagonist, no. 1361), A779 (AT1-7/MAS antagonist, no. 5937), HIF142 (AT4/IRAP antagonist, no. 5627). The final concentrations of both angiotensins were 1nM whereas antagonists were used at 1000 nM. Given that these peptides are relatively quickly degraded, the experimental medium was changed every 24 h.

### 4.2. Metabolic Activity Assay

We used colorimetric MTT Assay for assessing cell metabolic activity. The method involves reduction of tetrazolium dye 3-(4,5-dimethylthiazol-2-yl)-2,5-diphenyltetrazolium bromide (yellow) to water-insoluble formazan (purple) by mitochondrial dehydrogenase in viable cells. Details of this procedure have been described previously [9]. Ang-(1-9) and Ang-(3-7) was added to the culture medium at the final concentration ranging from 0.1 nM to 1000 nM. The combinations of both angiotensins (1 nM) with inhibitors (1000 nM): Losartan, PD123319, A779, HIF142 were also tested. Towards the end of the incubation period (24 or 48 h), a MTT was added to each well. The crystals formed by viable cells were dissolved in DMSO. Absorbance was measured using a BioTek ELX808 microplate reader with 570 nm filter.

### 4.3. The Cell Cycle Assay

The cell cycle profile was measured by flow cytometry based on the DNA content of cells. The Muse™ Millipore Cell Cycle Kit (Merck, no. MCH100106) comes with propidium iodide (PI), a red-fluorescent dye that binds to DNA, generally without a sequence preference. The somatic cells in the human body in G0 or G1 phases of the cell cycle are diploid (2n). During S phase, replication increases the DNA content of the cell from 2n to 4n. The subpopulation of cells in G2 and just prior to mitosis (M) contain exactly twice as much DNA (4n). The prostate cancer cells after 48 h incubation with experimental media (1 nM Ang or 1 nM Ang + 1000 nM inhibitor) were trypsinized, and a cell cycle assay was conducted according to manufacturer’s recommendations. The fluorescence was measured using a compact cytometry instrument Muse Cell Analyzer (Merck, Millipore, Burlington, MA, USA). The results are expressed as the fold change relative to control probes.

### 4.4. Migration Assay

We placed wound healing silicone insert (Ibidi, Fitchburg, MA, USA) with 4 defined cell-free gaps (500 µm) and one center gap (1000 µm) in each well of a 6-well plate. A cell suspension was placed in the wells at concentration 4–5 × 10^5^ cells/mL. After 24 h, when the cells created a confluent layer, the culture inserts were gently removed using sterile tweezers. Then 1 mL of medium with or without angiotensin (1 nM) was added to the each wells. The wound closure was documented by a series of photographs. The area of the wounded surface and its closure was measured by ImageJ software (Wayne Rasband, National Institutes of Health, USA; http://imagej.nih.gov/ij) at different times (0–48 h). The results were expressed as a percentage of initial wound area at time zero.

### 4.5. Soft Agar Anchorage-Independent Assay

The soft agar colony formation assay was used to investigate changes in the aggressiveness in the tested cells. This a well-established method to monitor anchorage-independent cell growth has been described previously in detail [13]. The cells were suspended in 0.3% low-melt agarose and were added to the top of the solidified base layer agar. Every 2–3 days, the cells were treated with another dose of the Ang-(1-9) or Ang-(3-7). After 11-14 days, the number of colonies in the semisolid medium and total area of colonization were counted using 0.005% crystal violet and ImageJ software (Wayne Rasband, National Institutes of Health, USA; http://imagej.nih.gov/ij).

### 4.6. RT-qPCR

The real time quantitative polymerase chain reaction (RT-qPCR) was used to detect the level of gene expression. The prostate cells were exposed to Ang-(1-9) or Ang-(3-7) at a concentration of 1 nM for 48 h. Total RNA was extracted from the cells using TRIzol reagent and purified with the phenol: Chloroform method. cDNA was synthesized from 10 μg of total RNA using ImProm RT-IITM reverse transcriptase (Promega, Madison, WI, USA). A RT-qPCR reaction was performed with LightCycler 96 (Roche, Basel, Switzerland) with 2 μL of cDNA. The details of the procedure including endogenous internal controls (*H3F3A* and *RPLPO*), annealing and detection temperature and primer sequences have been previously presented [13,15,49]. The experiment was conducted in, duplicate from three repeats. Differences in gene expression were calculated by REST-MCS beta software version 2 (2006) (www.gene-quantification.info).

### 4.7. Western Blot

RIPA Buffer (Sigma-Aldrich/Merck, Saint Louis, MO, USA) supplemented with phosphatase inhibitors (Sigma-Aldrich/Merck, Saint Louis, MO, USA) and proteases inhibitors (Sigma-Aldrich/Merck) was used for fast, efficient cell lysis and protein isolation. The protein concentration was assessed using the Direct Detect Infrared Spectrometer. This device uses infrared (IR) to quantify proteins in the sample. This is done by measuring amide bonds in protein chains. The sample does not require any previous preparations, it is not necessary to perform a standard curve. The details of the Western blotting procedure has been described previously [50]. The protein samples were mixed with Laemmli Lysis-Buffer and heated at 100 °C for 5 min. Denaturated proteins with mass 30 µg were separated in SDS-PAGE gel and transferred to nitrocellulose membrane (Sigma-Aldrich/Merck) using Mini-PROTEAN Tetra system (Bio-Rad, Hercules, CA, USA). The blocked membranes (5% non-fat milk in TBST buffer) firstly were incubated with primary antibodies: androgen receptor (Cell Signaling Technology, Leiden, The Netherlands no. 5153), estrogen receptor α (Cell Signaling Technology no. 13258S), estrogen receptor β (Thermo Fisher Scientific, Waltham, MA, USA no. PAI-311) and then with secondary antibodies conjugated with alkaline phosphatase (Sigma-Aldrich/Merck).The β-actin (housekeeping gene) (Cell Signaling Technology, Leiden, The Netherlands no. 8457S) constituted loading control to normalize the amount of loaded sample. The bands were visualized with Novex AP Chromogenic Substrate (Life Technologies, Corporation, Carlsbad, CA, USA). The densitometric analysis of protein level were measured using ImageJ software (Wayne Rasband, National Institutes of Health, USA; http://imagej.nih.gov/ij).

### 4.8. Statistical Analysis

The data are presented as mean ± SD of at least three independent experiments. The measurements were subjected to analysis of variance (One-Way ANOVA) and Dunnett’s or Tukey’s Multiple Comparison Test using GraphPad Prism 5 (GraphPad Software, La Jolla, CA, USA; www.graphpad.com). The accepted level of significance was *p* < 0.05.

## Abbreviations

Ang-(1-9)	Angiotensin 1-9	
Ang-(3-7)	Angiotensin 3-7	
RAS	Renin–angiotensin system	
BCL2	B-cell lymphoma 2	
T	Testosterone	
ACE1	Angiotensin Converting Enzyme-1	
ACE2	Angiotensin Converting Enzyme-2	
NF-kB	Nuclear Factor kappa B	
HIF1a	Hypoxia-inducible factor 1-alpha	
ROCK1	Rho Associated Coiled-Coil Containing Protein Kinase 1	
VEGF	Vascular Endothelial Growth Factor	
MAS	Proto-oncogene Mas	
AR	Androgen eceptor	
ESR1	Estrogen Receptor 1	
ESR2	Estrogen Receptor 2	
I1	Inhibitor AT1; losartan	
I2	Inhibitor AT2; PD123319	
I3	Inhibitor AT1-7/MAS; A779	
I4	Inhibitor AT4/IRAP; HIF142	
DHT	Dihydrotestosteron	
BIRC5	Baculoviral IAP Repeat Containing 5; Survivin	
BAX	Bcl-2 Associated X-protein	
MKI67	Marker Of Proliferation Ki-67	
AT1	Angiotensin Receptor Type 1	
AT2	Angiotensin Receptor Type 2	
AT4/IRAP	Angiotensin Receptor Type 4/Insulin-Regulated Aminopeptidase Enzyme	
E2	Estradiol-17β	
